# Parent views on the content and potential impact of respiratory tract infection surveillance information: semistructured interviews to inform future research

**DOI:** 10.1136/bmjpo-2017-000036

**Published:** 2017-08-11

**Authors:** Joanna May Kesten, Emma C Anderson, Isabel Lane, Alastair D Hay, Christie Cabral

**Affiliations:** 1 The National Institute for Health Research Health Protection Research Unit in Evaluation of Interventions, School of Social and Community Medicine, University of Bristol, Bristol, UK; 2 The National Institute for Health Research Collaboration for Leadership in Applied Health Research and Care West (NIHR CLAHRC West) at University Hospitals Bristol NHS Foundation Trust, Bristol, UK; 3 Centre for Academic Primary Care, School of Social and Community Medicine, University of Bristol, Bristol, UK; 4 NIHR School for Primary Care Research, School of Social and Community Medicine, University of Bristol, Bristol, UK

**Keywords:** qualitative research, infectious diseases, comm child health, respiratory, virology

## Abstract

**Objectives:**

This study explored the potential value of real-time information regarding respiratory tract infections (RTIs) circulating in the community by eliciting parent views on illustrative surveillance information and its possible impact on primary care consultations.

**Design:**

Semistructured interviews were conducted with parents of children (>3 months–15 years). Participants were presented with example information on circulating viruses, symptoms and symptom duration and asked about its potential impact on perceptions of child illness and management practices. Interviews were analysed using the framework method.

**Setting:**

Parents participating in a cohort study were selected purposefully using index of multiple deprivation and child age.

**Participants:**

30 mothers of children (>3 months–15years).

**Results:**

Parents anticipated using the information to inform lay diagnoses particularly when child symptoms were severe and thought normal symptom duration awareness might extend the time prior to seeking medical advice, but it also may encourage consultations when symptoms exceed the given duration. The information was not expected to change consultation behaviour if parents felt their child needed a medical evaluation and they felt unable to manage the symptoms. Most parents felt that the information could provide reassurance that could reduce intention to consult, but some felt it could raise concerns, by heightening awareness of circulating viruses. Lastly, parents wanted advice about protecting children from circulating viruses and felt that general practitioners using the information to diagnose child RTIs with greater certainty was acceptable.

**Conclusions:**

Diverse responses to the surveillance information were elicited, and there was some support for the intended outcomes. This study has important implications for the design of interventions to modify consulting behaviour. Future piloting to measure behaviour change in response to infection surveillance information are needed.

**What is already known on this topic?**

- Reducing paediatric consultation rates for respiratory tract infections could beneficially impact on primary care resources, antibiotic use and help address antimicrobial resistance.
- Primary care help-seeking decisions are informed by parent uncertainty, self-efficacy, perceived symptom severity and social norms.
- Parents want consistent, trustworthy advice to support home management of child respiratory tract infections and guidance about when to seek medical advice.

**What this study hopes to add?**

- This study demonstrates the importance of qualitative research for developing intervention content and understanding the acceptability of an intervention.
- Parents liked online infection surveillance information; typical symptoms and symptom duration information appear likely to have an impact on uncertainty, concern and consultation intention.
- Future research is needed to develop systems to support the provision of consistently available online infection surveillance information at the community level.

## Background

The increasing threat of antimicrobial resistance (AMR) is a significant public health problem largely attributable to the overprescription and misuse of antibiotics.^1–5^ Prescriptions for respiratory tract infections (RTI) in primary care account for 60% of all antibiotic prescriptions.^6^ RTIs are the most common problem managed by primary care,^4^ and the majority of these infections occur in children.^7^ Consultations for paediatric RTIs have a significant cost burden^6 8^ and often end with an antibiotic prescription despite no clinically significant impact on recovery time^9^ and infections being largely self-limiting. A small change in consultation rates for paediatric RTIs could have a significant impact on primary care resources, the use of antibiotics and help reduce the growing threat of AMR.

Several factors inform parent primary care help-seeking decisions.^10^ Parent uncertainty^10^ and self-efficacy in distinguishing between serious and ‘normal’ self-limiting illness and in the ability to manage the illness at home influence help-seeking decision making.^11^ Perceived severity is influenced by symptoms lasting longer than expected and impact on eating, drinking and sleeping.^11 12^ Social norms are also important^13^; consulting is perceived to be the safest course of action when there is uncertainty regarding symptom severity and seriousness.^13^ Consulting reduces parent uncertainty by providing a medical evaluation, reassuring parents that the illness is self-limiting and advising on appropriate treatment, symptom relief and ways to prevent future illness.^11 12^ Parents want consistent, trustworthy advice to support home management of RTIs and help regarding when to seek medical advice.^10 11^ Currently, parents do not feel this type of support is available outside of a primary care consultation.^11^

Online resources could provide parent help-seeking support and could include community-based, real-time circulating RTI and symptom duration information. They could address some of the aforementioned factors. We hypothesised that online information about currently circulating viral illnesses, with symptom and home care advice, could decrease parental anxiety and encourage home management of RTIs, leading to reductions in primary care consultations.^14^ The Evaluation of Enhanced Paediatric Respiratory Infection Surveillance (EEPRIS) Study,^14^ conducted by members of this team, has recently assessed the feasibility of collecting such community-based real-time syndromic and microbiological RTI surveillance data (unpublished). In addition to establishing the feasibility of collecting RTI surveillance information, and in preparation for intervention development, it is pertinent to use qualitative methods to understand the ‘in-principle’ acceptability of an intervention to the target group before progressing to trial.^15 16^ Sekhon and colleagues recently theorised the concept of acceptability as ‘A multi-faceted construct that reflects the extent to which people delivering or receiving a healthcare intervention consider it to be appropriate, based on anticipated or experienced cognitive and emotional responses to the intervention’ (p. 4).^17^ Understanding potential responses to new interventions is an important means of developing and enhancing intervention appropriateness, feasibility, influence and engagement.^15 16^

We therefore explored parent views on the content, and the potential impact on RTI management, of locally relevant, real-time infection surveillance and RTI information to inform the development of a future intervention.

## Methods

### Online infection surveillance information development

**Figure 1:**
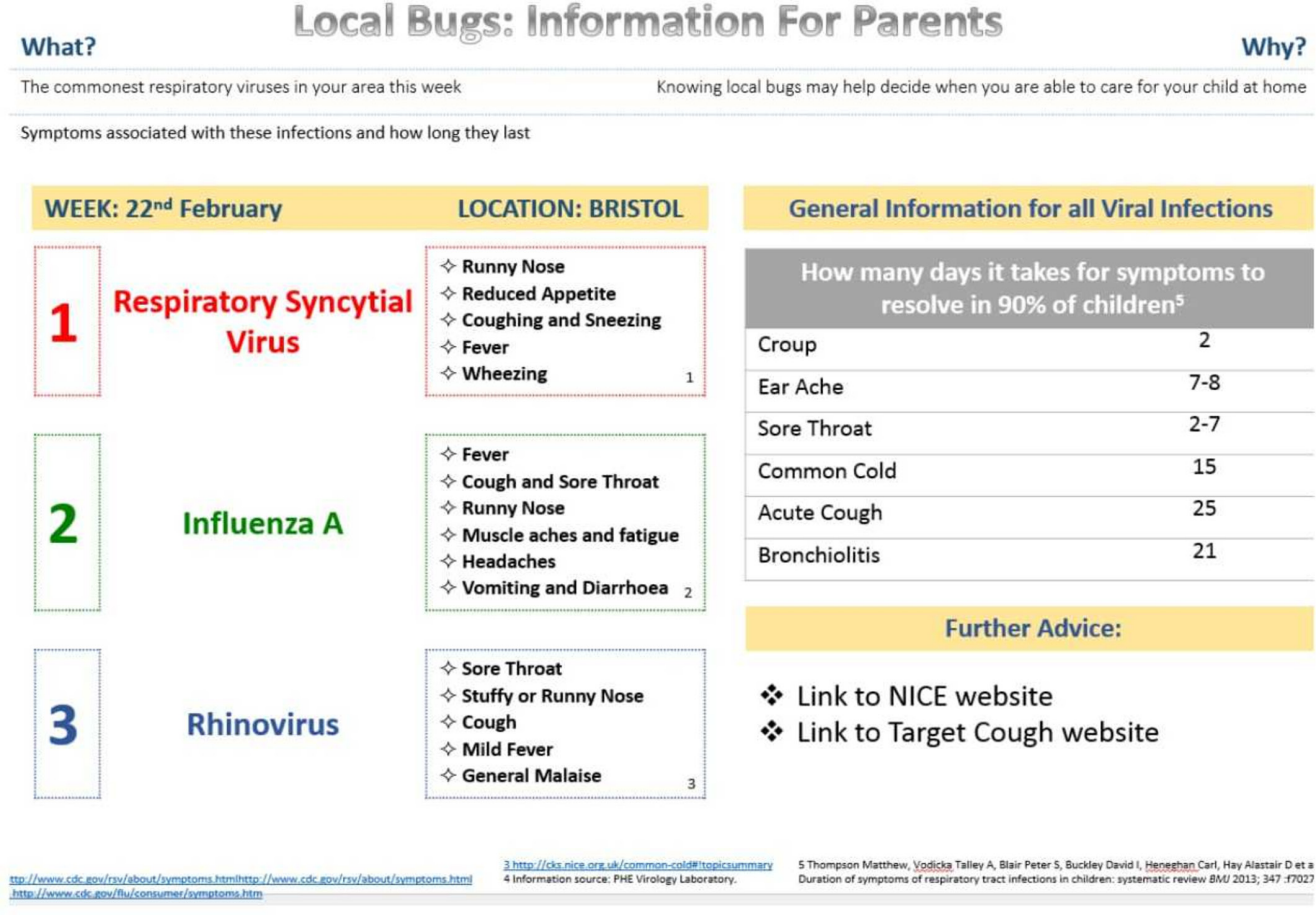
Online surveillance information version 1.

**Figure 2:**
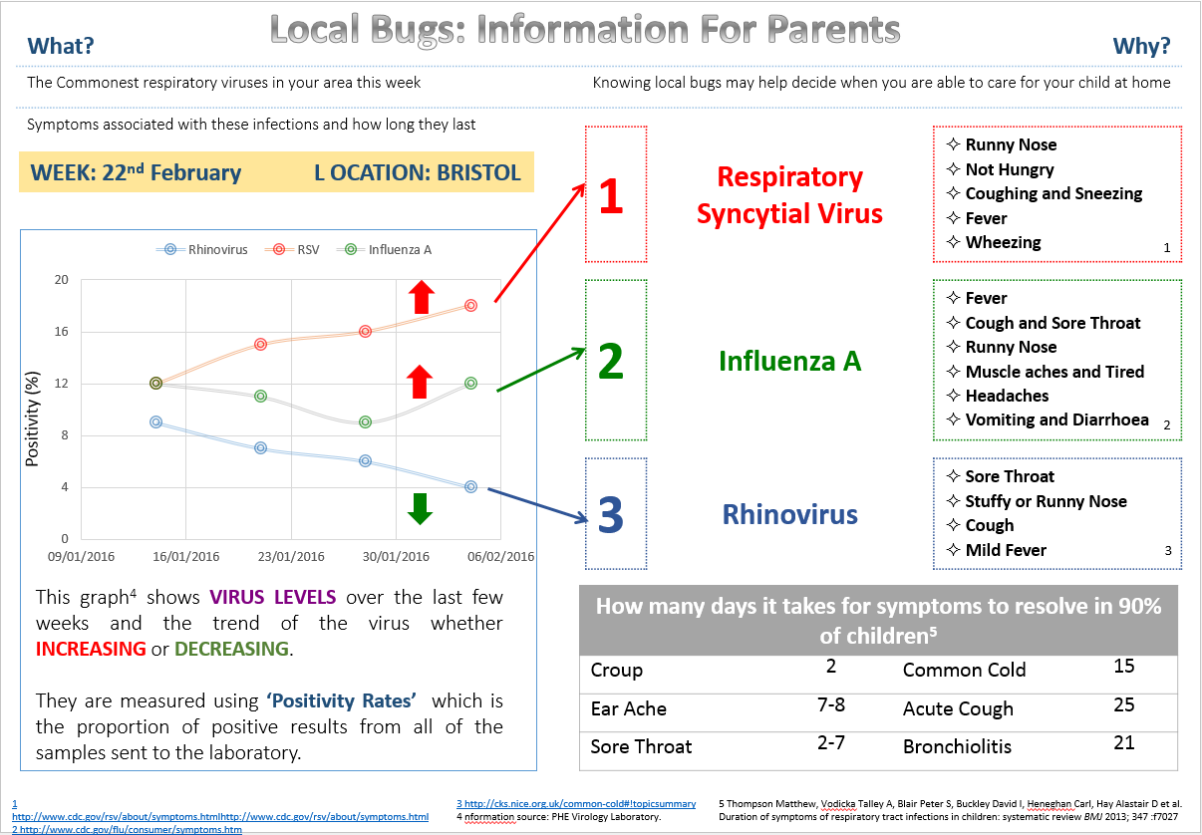
Online surveillance information version 2.

Two examples of online infection surveillance information (figures 1 and 2) were developed with example viral RTI data. We opted to focus on viral RTIs since the evidence that these are associated with primary care RTI presentations is stronger than for bacterial infections.^18 19^ The information contained a brief introduction, three prevalent viruses, their symptoms and common clinical presentations^20 21^ and the number of days it takes for common viral symptoms to resolve in 90% of children.^22^ Version 2 also contained a graph of positivity rates of commonly circulating viruses over time.^23^ Members of the EEPRIS study team, including primary care clinicians (ADH and IL), contributed to the design of the information. Feedback from a patient and public involvement group of parents was used to further develop the information in advance of commencing interviews.

### Study design and participant recruitment

Semistructured face-to-face interviews were conducted by JMK with parents participating in the EEPRIS study, which recruited children (≥3 months–15 years) and their parents/carers via general practice surgeries in Bristol.^14^ To achieve a sample with maximum variation in views, we used a purposeful sampling approach. All EEPRIS parents who provided written informed consent for an interview were eligible for inclusion. Parents were invited to participate using an emailed or mailed information sheet and were selected based on socioeconomic status (index of multiple deprivation (IMD) decile using home postcode) and age of child (<7 years or ≥7 years) who had reported symptoms to the EEPRIS study.^14^ Three parents had not reported any symptoms to the EEPRIS study and were recruited to maximise diversity of the study. Parent gender was not a characteristic used to sample participants for interview. One father was invited to participate out of a potential 27 or 9% of the EEPRIS cohort.

If a potential participant did not respond to the invitation and follow-up telephone call or declined to participate, an invitation was sent to another parent with similar characteristics. Interviews were conducted at a convenient time for the parent and in their home and were audio recorded using an encrypted recording device, transcribed in full and verbatim, checked for accuracy and anonymised. Participants received a £5 shopping voucher as a thank you for their time. Interviews continued until theoretical saturation of key concepts had been reached and little new information emerged.^24^

### Interview process and topic guide

Parents were presented with both versions of the information on paper (figures 1 and 2) and were informed that parents and general practitioners (GPs) would be able to access it. Parents were asked to imagine how they might use the information if their child has the symptoms of a RTI (online appendix A). Using paper website ‘prototypes’ for this purpose has been recommended previously.^16^ Parents were also presented with hard copies of screenshots of the ‘Caring for Children with Coughs’ website developed by researchers at the University of Bristol including members of the EEPRIS team.^25^ The aim of this website is to support parents to care for their child when they have a cough. It was explained that this type of information would accompany the surveillance information. Parents were not asked to comment on this information.

10.1136/bmjpo-2017-000036.supp1 Supplementary file 1

Interviews began by discussing the acceptability of the EEPRIS feasibility study (interview topics and findings to be reported separately). Parents were then asked to discuss their usual approach to RTI management (eg, factors influencing medical advice seeking) (not included in the current analysis) (online appendix A). The interviews then explored the perceived value and impact of receiving the surveillance information including its usefulness and potential impact on perceptions of child illness and management practices such as intentions to consult. It is recommended that to increase the likelihood of effectiveness, interventions are developed using relevant theory.^26^ The interview topic guide was informed by a hypothesised behaviour change pathway developed using the components of the Capability, Opportunity, Motivation, Behaviour (COM-B) model^27^: physical capability, psychological capability, social opportunity, physical opportunity and motivation. The COM-B model was chosen for this purpose as it offers a systematic approach to considering key factors acting on the behaviour of interest, in this case visiting the GP for child RTI. Feedback on the content and presentation was also sought. The interview guides were applied flexibly to allow for emergent issues to be probed.

### Analysis

Gale and colleagues’ framework method, a type of thematic analysis, was used to analyse the data.^28^ This method was chosen because by condensing and summarising the data within a framework matrix, reflections on meaningful, pertinent themes as well as connecting or divergent perspectives were formed. Analysis began with a process of familiarisation with the transcripts during which initial impressions were noted. Two researchers independently assigned codes to the first three transcripts systematically line-by-line, which summarised and interpreted the data. Initial codes were discussed among the study team, iteratively refined and condensed into broader themes to produce an agreed coding framework that was applied to all subsequent transcripts. Throughout this coding, modifications were made to the framework in response to new emergent information. The coded data were then inserted into a framework matrix in QSR NVivo V.10, which charted the themes against each participant. Within the matrix, the meaning in the data was summarised.

### Ethics

Ethical approval for this study was granted by the South West Frenchay Bristol Research Ethics Committee (reference: 15/SW/0264). Written informed consent was sought prior to interview.

## Results

Thirty mothers were interviewed out of 58 invited (52% recruitment rate) (table 1). Only one parent actively refused to participate; the remaining parents did not respond to the invitation and could not be contacted by telephone. The interview length averaged 44.8 min (range 31.3–64.4 min).

**Table 1:** Parent interview participant characteristics

Characteristics	n
Parent gender	
Female	30
Age of child used to select parent	
Preschool (<4 years)	16
Primary school (4–11 years)	10
Secondary school (>11 years)	4
Number of children per family	
1	14
2	15
3	1
Index of multiple deprivation decile (1=most deprived, 10=least)	
1–3	8
4–6	6
7–10	16

### Perceptions of online infection surveillance information

#### Parents were interested in circulating virus symptoms and symptom duration

Most parents thought information on symptom duration and locally circulating viruses and their symptoms was useful for them. Interest in locally circulating viruses was described by one parent as stemming from parents’ ‘morbid fascination’ with child illness. Few parents had been previously aware of expected symptom duration; indeed, some were surprised by how long symptoms last. In contrast, one parent felt that interpreting symptom duration information for multiple simultaneous symptoms was confusing, and another preferred not knowing what viruses her children may catch. A small number of parents commented that this information would be particularly relevant to first-time parents and parents of young children and, those with pre-existing immune system conditions.

*It would be really useful, I think, to know more about what viruses are circulating, so if that contributes towards that, that’s great.* (Interview 22, IMD decile 8, child age 4 years 2 months)

*Maybe [for] a new mum you know or a mum to young children, I think that’s handy.* (Interview 29, IMD decile 4, child age 13 years 1 month)

### Likelihood of accessing online surveillance information

Most parents anticipated looking at the information if it was available. Roughly half of parents would not use the information if their child’s symptoms were perceived to be mild (eg, symptoms of a common cold) but would consider using it for more severe symptoms, such as vomiting, diarrhoea and bronchitis, with prolonged symptom duration, impacting on the child’s activities and/or are getting worse. Unlike mild symptoms, which parents felt confident managing, these symptoms were perceived to be more worrying for parents and were anticipated to be more likely to require information seeking to inform some form of action, for example, alleviating child symptoms or seeking medical advice. A couple of parents said they would not use the information daily, but one said they would check the website frequently to monitor changes. In contrast, one parent felt they did not need to access the information because they felt confident managing child illness, and a small number thought the information was interesting and they would look at it, but did not know how they would use it. A small number of parents said they would use the information in conjunction with the NHS Choices website. Information trustworthiness was viewed as important by a small number of parents.

*If one of them became more unwell than you know, just a bit of a runny nose, and had a bit of a temperature or was wheezing, headaches, vomiting, diarrhoea, some of the symptoms you’ve got in here, I might look here first.* (Interview 8, IMD decile 8, child age 3 years 4 months)

### Potential impact and usefulness of online infection surveillance information

#### Lay diagnosis and consulting

Most parents anticipated using the information to inform a lay diagnosis by matching their child’s symptoms to the circulating viruses. However, a small number of parents felt uncertain about diagnosing accurately from the current information. If a child’s symptoms do not match those listed for circulating viruses, again a small number of parents predicted feeling justified in consulting due to resulting increased concern.

*If something else is there for your child, there is something extra signs and symptoms is there, and then that can be a worry as well - what else is there? (…) ‘Oh my God, am I needing to go to the GP and ask them what it is?’ (…) Or must be the same virus, but your child just may affect a bit more - you don’t know, do you, really?* (Interview 5, IMD decile 3, child age 3 years 10 months)

*All you’re really doing is helping them mis-diagnose their child, I think, because it’s not going to be 100% certain that even if she were to check all those, that it’s definitely that, without her seeing a doctor.* (Interview 1, IMD decile 5, Child age 2 years 6 months)

#### Context-specific anticipated impact on management of child illness

Symptom duration information was anticipated to help parents feel more prepared to cope with child illness in the context of daily commitments (eg, work, childcare and school attendance). Awareness of symptom duration may also extend the time prior to seeking medical advice or prevent consultation; however, parents felt that if their child’s symptoms exceeded the duration given in the online information, this may encourage consultation.

*Knowing how long the lifespan of it is is quite useful I think, sort of plan your life a bit then!* (Interview 19, IMD decile 9, child age 2 years 10 months)

*I wouldn’t maybe go to the GP or I would wait longer unless you know because as I say I tend to worry after a long time so maybe it’s not that but maybe I would give it a bit longer.* (Interview 13, IMD decile 6, child age 10 years 9 months)

If parents felt their child needed to be seen by a healthcare professional and they were concerned or felt unable to manage the symptoms at home, then this information was not expected to alter usual consultation behaviour. Indeed, several parents noted that the information did not advise how to care for children with a circulating virus.

*I don’t know whether that is going to change my behaviour in taking them to the GP’s if they are unwell and if I can’t manage them.* (Interview 5, IMD decile 3, child age 3 years 10 months)

*It says here knowing local bugs may help decide you when you’re able to care for your child at home, I don’t know quite how that is the case in the sense that it tells you what something is, but that doesn’t tell me how serious it is, or whether or not I need to go and see a doctor.* (Interview 9, IMD decile 7, child age 11 months)

#### Reassurance and concern

The majority of parents felt that awareness of circulating viruses could lead to reduced concern and increased reassurance by facilitating lay diagnosis. Parents anticipated that awareness of others experiencing similar symptoms would be reassuring. Knowing how long the symptoms are likely to last was believed to help parents perceive their child’s symptom duration as normal. Some parents also commented that these factors could reassure parents that the decision *not* to consult was appropriate. Alternatively, some parents felt the information may not have any impact on their levels of concern.

*Re-assurance, okay it’s not just us which I think is comforting in a weird kind of way.* (Interview 28, IMD decile 1, child age 1 year 6 months)

Parents felt the information could also raise their concern, for example, where symptoms last longer than suggested by the symptom duration information and by heightening their awareness of circulating viruses that children *could* catch. The latter relates to parents’ concerns about the threat posed by circulating viruses leading to a desire to protect children by limiting interactions with others. An additional source of concern for parents was the use of medical names (eg, *Rhinovirus* a*nd croup*) that were perceived as serious. The information may therefore encourage parents to label and perceive their child’s symptoms as more serious than they would have otherwise. In contrast, two parents commented that the information does not provide an indication of symptom severity and that they would respond to the severity of their child’s symptoms.

*I think it could have been actually a negative thing ‘cause I think it’s out of your control where your child goes and what they catch and are you just gonna be sitting there thinking ‘oh my god! This is going around, don’t let them out!’* (Interview 19, IMD decile 9, child age 2 years 10 months)

*You would be a lot more worried if you thought it was Bronchiolitis when you originally just thought it was a cold.* (Interview 23, IMD decile 8, child age 2 years 3 months)

#### Prevention of illness

Parents wanted to know how to protect their child from circulating viruses and anticipated increased vigilance and encouragement of hand hygiene and limiting interactions with others to prevent infection.

*We tell them you know, ‘Be careful when you go to school. Wash your hands that way. Don’t touch here and there. The tissues that you use, put them in the bin,’ so they don’t keep hold of it, and that kind of little everyday things you can tell the children, if you know that there is something going around.* (Interview 5, IMD decile 3, child age 3 years 10 months)

### Acceptability of the use of online infection surveillance information in consultation

Parents approved of GPs using the information in part due to their common experience of online information sharing during consultations. Parents felt the information could also help them identify the most important symptoms to report during consultations. GPs sharing this information within consultations were expected to reassure parents and support more accurate diagnosis. Indeed, some parents felt the information would be more useful for clinicians than parents. This may relate to the finding that a small number of parents did not know how they would use it.

*To know the things that it’s important to mention to the GP as well if they’ve got a whole range of symptoms, obviously you want to try and go through all of them but knowing you know, what potentially is involved, what sets this illness apart or makes it different to something else.* (Interview 25, IMD decile 8, child age 1 year 7 months)

*I think that’s really useful, because for a GP, it must be difficult for them to make a diagnosis with anything like coughs and colds and this, that and the other, so I would have thought it’s useful for them to have the information. (…) I think it would be, like, reassuring, I suppose, that they were able to use the information to make, probably, more of an accurate … not necessarily a diagnosis, but more of an accurate guess as to what it’s likely to be.* (Interview 23, IMD decile 8, child age 2 years 3 months)

*I don’t think we necessarily need - as parents or members of the community - we don’t necessarily need to see all this detail…this is probably useful for you and GPs maybe but I think as a snapshot this is useful to know.* (Interview 10, IMD decile 4, child age 7 years 11 months)

### Design feedback and improvements

#### Information clear but too complex

Several parents perceived the design of the information positively, commenting that it was clearly presented. However, for a few parents, the information was too complex and difficult to interpret. For example, unfamiliar virus names (eg, Rhinovirus), positivity rates shown in the graph and ranking of the top three viruses were unclear. In addition, a few parents commented that the information should clarify whether children need to have all the listed symptoms to have the circulating virus or whether the same virus could present with fewer or different symptoms. Parents also felt that the information needed to be converted into lay language (eg, common name and description of circulating virus).

#### Interpreting viral infection information

Only a small number of parents understood that by identifying these infections as viral, the information was trying to convey the message that these infections do not require antibiotic treatment. Similarly, two parents wanted the information to state whether the circulating illnesses require antibiotics and one parent felt that improvements to the information were needed to help inform parents of the differences between viral and bacterial infections. One parent suggested that the information could be modified to highlight the symptoms of bacterial infections to help parents deduce whether their child’s symptoms are likely to be viral or not.

*I know these are all viruses so you can’t do much about them in terms of medicines.* (Interview 13, IMD decile 6, child age 10 years 9 months)

*That might be helpful to say actually if there’s none of these signs then it’s probably a virus and we won’t give you any treatment so that’s just reassuring.* (Interview 24, IMD decile 8, child age 2 years 5 months)

#### Preference for information without the graph

The majority of parents preferred the information without the graph, which was described as confusing, complicated, requiring too much interpretation and risking misinterpretation. For some, the graph was more information than parents need because they are primarily concerned with the child’s current symptoms rather than trends over time. A small number of parents noted that the information in the graph could be misinterpreted as representing all children in the area rather than a sample of unwell children. In contrast, some parents found a visual representation useful and a small number interpreted changes over time as an indication of how concerned they should be, stating that they need to be more aware of increasing positivity rates as their child may be more likely to catch these viruses.

*It does take you a while to understand what the graph is actually saying.* (Interview 12, IMD decile 7, child age 11 years 4 months)

*[The graph is] kind of handy actually because if you look at something like influenza A then you can see oh there is a bit of a rise locally, you have gotta be a bit more aware.* (Interview 18, IMD decile 8, child age 3 years 6 months)

#### Parents wanted information that was more relevant to their context and needs

Parents felt the information would feel more relevant if it was personalised to their local area (home postcode, school catchment area or north and south of the city), presented on a map, with child age and symptom checker technology.

Most parents also wanted the information to include specific advice on how to treat and manage the symptoms of the circulating viruses and when to consult.

*It would be more relevant if it was narrowed down around school.* (Interview 10, IMD decile 4, child age 7 years 11 months)

*I sort of felt like that (a map) might be a bit more visually accessible maybe than the graph.* (Interview 15, IMD decile 4, child age 3 years 0 months)

### Accessing online surveillance information

Parents highlighted the importance of the information being easily accessible and well advertised, stressing that they would be unlikely to actively look for it. To promote the information, written and verbal channels of communication in healthcare, education and childcare settings, on the internet and mobile phone apps were suggested. Informal information sharing among parents was also reportedly common and could be encouraged.

## Discussion

### Principal findings

This study found that the proposal for online infection surveillance information was liked by parents, and they felt clinicians’ use of the information during consultations would be acceptable. The views of the potential uses and impacts of the information were diverse and complex. While symptom information was felt likely to inform lay diagnosis of child symptoms by parents, there remained uncertainty about whether the diagnoses would be accurate and whether it would reassure or raise concern. Parents felt the symptom duration information had potential to extend the time prior to seeking medical advice or avoid consultation altogether by reassuring them that durations were normal. A few parents felt that simply knowing their child’s symptoms matched those of a locally prevalent virus would be reassuring. However, some parents felt that the information may increase uncertainty, concern and consultation intention if the symptoms did not match the circulating RTIs, symptom duration exceeded the online information and/or parents felt the information was identifying illness that were potentially more serious (eg, *bronchiolitis*). In situations where parents felt their child needed to be seen by a healthcare professional and were concerned or felt unable to manage the symptoms at home the information was not expected to influence consulting decisions.

### Strengths and limitations of the study

We achieved a diverse sample of mothers in relation to opinions, socioeconomic status and child age. The sample was limited to mothers; however, research has shown that they are more likely to take children to consultations than fathers.^29^ It should be noted that the EEPRIS cohort cannot be considered representative of all parents, parents had higher levels of educational attainment, resided in less deprived neighbourhoods and with younger children than the invited population (unpublished findings). Although the sampling approach for this qualitative study did not produce a similar number of responses across the categories of child age and deprivation, the sample closely reflects the EEPRIS cohort (unpublished findings). The recruitment rate for this study was high suggesting that the parents were interested in the topic and participation in the cohort study supported participation. Given the range of viewpoints, we expect our findings to be transferable beyond the EEPRIS cohort. Although the lead researcher (JMK) emphasised that she had not developed the information, there is the potential for response bias towards positive feedback. It should also be stressed that we have elicited hypothetical responses to a potential intervention, which means future piloting to measure behaviour change is needed. In the next stage of the EEPRIS study, these findings will be used to develop a comprehensive logic model of the hypothesised intervention effects and behaviour change techniques.^30^ An online randomised experimental study will assess the efficacy of the next iteration of intervention material and the related behaviour change techniques.^30^ In the next iteration, the graph of positivity rates has been replaced by a map of the local area on which the prevalence of common viruses is visualised, specific self-care instructions relating to viral infections is provided and the distinction between viral and bacterial infections is described. Short and simple messages are used, and there is repetition of key points. Finally, the information highlights that it comes from a credible academic source and was cocreated with parents.

The novelty of the intervention is a strength, and the findings from this study have important implications for the design of interventions aiming to modify parental consulting behaviour.

### Comparison with existing literature

In line with previous research, these findings suggest that online surveillance information may go some way to meeting parents’ information needs.^10 11^ The development of online, accessible^10^ information interventions is supported by previous research that demonstrates that parents use a variety of information and advice sources including websites.^11^ Review evidence supports the provision of information on circulating viral illnesses rather than general information about antibiotic overuse and resistance.^31^ Indeed, clinician diagnosis of a general ‘virus’ is viewed as ‘unsatisfactory’ by parents as it does not provide a specific diagnosis^10^ and is seen as ‘trivialising’ parent concern^12^; supporting parents’ to make a lay diagnosis may help address this issue.

Only a small number of parents understood that the information on viruses was attempting to convey the message that the infections do not require antibiotic treatment. The fact that few parents felt this information would influence their consulting decisions could mean that the information needs to state this more explicitly. Alternatively, it could mean that parent decisions to consult are driven by a range of factors, not just perceived need for antibiotics. Previous findings suggest that parents are driven to consult by a desire to remove potential health threats despite recognising that the risk of this threat is low.^13^ Kai and colleagues also found that parent beliefs about the use of antibiotics are informed by illness severity and impact on the child, not whether the illness is viral or bacterial.^10^

The suggestion in this study that first-time parents or those with younger children may find online surveillance information more useful than more experienced parents is supported by previous qualitative work, which has shown that less experienced parents have difficulties delineating between serious and minor coughs.^11 13^ Therefore, interventions like this may be most beneficial if targeted at first-time parents with young children.

The suggestion that some parents may use this information as part of their consultation decision-making process supports the findings of a systematic review that reported that interventions to influence consulting behaviour and antibiotic use for children with RTIs delivered prior to child illness compared with during consultations may be more effective.^31^ In turn, by supporting consultation decision making, parents may experience increased self-efficacy to manage child illness.

Given that prolonged symptom duration predicts consultation,^32^ influencing what is considered prolonged may influence consultation decision making. Previous experience of RTI management can also inform future management practice, either increasing or decreasing the likelihood of consultation intentions^11 13 31^; therefore, if symptom duration combined with surveillance information is able to extend time to consultation, or prevent consultations, then this may create a ‘virtuous cycle’ of reduced primary care attendance, reduced antibiotic use and reduced medicalisation of self-limiting illness.^31^ For example, symptom resolution during this time could reduce future consultation frequency.

Parent responses indicated that management practices are informed by perceptions of the severity of their child’s symptoms; however, the current examples of the surveillance information did not indicate the severity of circulating viral symptoms. Given that parent-rated illness severity is associated with self-reported consultation behaviour,^32–34^ additional information describing the (perceived) severity of circulating viral illness could help inform parents’ management practices. This study found that online surveillance information is unlikely to override parent intentions to consult if they are uncertain about child illness. Thus, although the current findings demonstrate some support for reduced uncertainty surrounding symptom duration and lay diagnosis, it is unlikely to remove all uncertainty. This is positive as it suggests that the information may not prevent consultations when they are needed. The notion that infection surveillance information could increase parent concern about the threat of infection relates to social norms of depicting children as vulnerable and the parent’s role in protecting children from harm.^13^

In line with previous research,^10 11^ we also found that parents wanted to know how to prevent RTIs and anticipated using the surveillance information to take preventative actions (eg, increased hand hygiene).

### Recommendations for future intervention design

This project illustrates the importance of qualitative research for exploring the acceptability of an intervention and developing intervention content with the population of interest.^15 16^ As far as possible, lay language should be used and information should be presented clearly to minimise the amount of interpretation needed. Typical symptoms and symptom duration appear likely to have an impact on uncertainty, concern and consultation intention. Advice on how to treat and manage symptoms, when to consult and how to prevent infection is wanted by parents. It is important for interventions such as these to consider all causes of parent concern relating to RTI management and to consider factors leading to consultation and uncertainty about self-care at home.

Another important area for future research is to develop systems to support the provision of consistently available online infection surveillance information at the community level. The online infection information in this study was based on microbiological data only. Future work could explore the potential use of other data sources such as syndromic/consultation data from GP electronic health records, which could be combined with microbiological data. A key challenge will be ensuring outputs are: sufficiently sensitive (to the rapidly changing epidemiology of infectious diseases), precise (based on large enough data sets) and specific (relevant to that geographical area).

## Conclusion

These findings indicate that online infection surveillance information and symptom duration information may be relevant to, and used by, parents. Diverse responses to the information were elicited, and there was some theoretical support for the intended outcome of reduced consultation intentions that in practice might lead to a change in consultation rates. For some parents, the information may reduce uncertainty and provide reassurance that could influence intentions to consult. Even small changes in consultation rates could have a significant impact on primary care resources and help reduce AMR.
