# Supplementary material for: Parent views on the content and potential impact of respiratory tract infection surveillance information: semistructured interviews to inform future research
Source: BMJ Paediatr Open. 2017 Aug 11;1(1):e000036. doi: 10.1136/bmjpo-2017-000036 (PMC5843006; doi:10.1136/bmjpo-2017-000036)
Supplement: Supplementary file 1 [file bmjpo-2017-000036supp001.pdf]

Appendix A. Interview topic guide

1. Introduction

Explain purpose of research project

Before we begin I'm going to give an overview of the two purposes of this interview study. We want to gather opinions on a possible future online resource of 'real-time' illnesses circulating in the community. We will use the information collected in the interview study to feed into the design of this online resource.

Explain audio recording procedures

I'd like to tell you that I will be recording the conversation to help us remember what we discussed. You can ask for the recording to be stopped at any time. What you say will be kept confidential and anonymous.

Ensure participant has a copy of participant information sheet

Answer any questions

Commence audio recording

1. Usual approach to RTI management

Before we discuss the online resource I'd like us to talk about how you usually care for your child when they have coughs, colds, sore throats, chest infections or ear infections. These are collectively called respiratory tract infections.

How do you usually look after your child when they have a respiratory tract infections?

What things influence whether you look after your child at home rather than take your child to visit a GP or other healthcare provider?

What things influence whether you take your child to visit a GP or other healthcare provider?

How easy or difficult do you find deciding when to take your child to a GP or other healthcare provider?

PROMPTS

Physical capability

Parent fatigue

Psychological capability

Knowledge/understanding of AMR

Knowledge of infections & key symptoms (i.e. what to look for in sick child)

Self-care knowledge

Social opportunity

Real or perceived pressure/expectations from others to visit GP

Real or perceived norms about other parents visiting GP

Antibiotic prescription provided previously for similar symptoms

Physical opportunity

Care alternatives/requirement to work or care for other children

Requirement for child to be attending school/other commitments

Availability of self-care tools (e.g. medication, thermometer)

Motivation

Habit of visiting GP for similar symptoms

Anxiety about child being ill/failing to recognise serious illness

Desire to ease child's symptoms

Desire to be back at work/other commitments

Attributing recovery from previous self-limiting illnesses to antibiotics

Beliefs about AMR

Perceived risk of serious consequences if GP advice not sought

Belief that duty to care to one's own child outweighs duty of care to the greater good

Trust in online information

Not wanting to waste the GPs time – fear of looking silly for taking child to GP

Where do you seek information or guidance on how to look after your child when they have an respiratory tract infections?

PROMPTS

Online information

Friends and family

Healthcare professionals

For what reasons do you choose these sources of information?

Is there any other information you would like to know when your child has a respiratory tract infections which is currently unavailable?

2. Perceived value and acceptability of online intervention

The second set of questions are about a possible future online resource of 'real-time' viral illnesses circulating in the community which parents and GPs could access. You may be given access to the online resource using a log-in username and password. Alongside this information researchers at the University of Bristol have developed a website to help parents

*care for their child when they have a cough including when to see a doctor and how to care for your child. *Refer to "Child-Cough.bristol.ac.uk"**

GIVE PARENTS TIME TO READ THROUGH PAPER COPIES OF THE INFORMATION AND ASK QUESTIONS.

What are your initial impressions of the information in this resource?

PROMPTS

Level of detail

User-friendly

Credibility/trustworthiness of information

What do you think is good/useful about this information? Can you describe your reasons for this view?

What do you think is not so good/bad about this information? Can you describe your reasons for this view?

PROMPTS

Symptoms (including severity and length) – are you surprised about the duration of illnesses?

Area level specific information

Advice about when to consult for various symptoms

How do you think the information could be improved or made clearer?

Is any of this information new to you? Which parts exactly? What have you learnt from this information that you didn't already know?

How do you think this information could be best shared with parents?

PROMPTS

Website

Social media e.g. Facebook

Schools/nurseries

GP emails

App for mobiles/ tables

Alerting system (e.g. sign-up to receive text messages)

Via GP vs online

3. Perceived impact of online intervention

Before this online resource is made available we would like to find out your views on how you might use the information, and what impact it might have on how concerned you are and how you look after your child when they have a RTI. During these questions I'd like you to imagine how you might use this information if your child has the symptoms of a RTI such as a sore throat, blocked or runny nose, sneezing and a cough.

How likely are you to use this online information when your child has an respiratory tract infections?

For what reasons/under what circumstances do you think you would look at/use this information?

For what reasons/under what do you think you would not look at/use this information?

PROMPTS

Out of curiosity / when child doesn’t have RTI symptoms

Child has RTI symptoms

Lack of knowledge about RTIs currently circulating and associated symptoms

Anxiety around cause of child’s symptoms and severity of illness

Lack of knowledge/understanding of infections & key symptoms (i.e. what to look for in sick child)

Lack of self-care knowledge

Unsure about need to visit GP

Complexity of information

Poor internet

Prefer to trust gut instinct

How would you use this information?

PROMPTS

Compare child’s current symptoms to those of circulating viruses

Set time limit in which to visit GP based on likely length of symptoms

Assess need to visit GP

What impact (if any) do you think this information might have on how concerned you might be about your child’s illness?

How do you think this information might influence how concerned you might be about the cause of your child’s symptoms and severity of illness?

Does this information make (illness described in scenario) seem more or less severe than before reading this information?

What impact (if any) do you think this information might have on what you do to manage your child's illness?

PROMPTS

Self-care at home

Visit GP

How would you use this information to decide whether to take your child to a healthcare professional such as the GP?

What things would encourage you to act in this way as a result of receiving the online resource?

What things would discourage you from acting in this way as a result of receiving the online resource?

How would you feel if a GP showed you this information during a consultation?

4. Close

That is the end of my questions. Before we finish is there anything I haven't covered today that you would like to add?

End of audio recording. Thanks for participating and give voucher.
